# Tracking of cardiovascular risk factors from childhood to young adulthood — the Pune Children's Study^☆^

**DOI:** 10.1016/j.ijcard.2014.04.105

**Published:** 2014-07-15

**Authors:** Suyog M. Joshi, Prachi A. Katre, Kalyanaraman Kumaran, Charudatta Joglekar, Clive Osmond, Dattatray S. Bhat, Himangi Lubree, Anand Pandit, Chittaranjan S. Yajnik, Caroline H.D. Fall

**Affiliations:** a Kamalnayan Bajaj Diabetology Research Centre, King Edward Memorial Hospital Research Centre, Pune, India; b Persistent Systems Ltd., Pune, India; c Medical Research Council Lifecourse Epidemiology Unit, University of Southampton, UK; d Department of Paediatrics, King Edward Memorial Hospital Research Centre, Pune, India

**Keywords:** Tracking, Cardiovascular risk factors, Childhood, Young adults, India

To target preventive measures appropriately in early life, it is important to know what childhood cardiovascular (CVD) risk profiles mean in terms of predicting adult risk. Studies from high-income countries have reported tracking of individual risk factors from childhood to adulthood [1–7]; there are no reports of child–adult tracking in low- and middle-income countries (LMICs). The Pune Children's Study is a cohort of 477 individuals born in the KEM Hospital, Pune, India in 1987–1989. We measured a range of CVD risk factors at 8 [8] (1996–7) and 21 (2009–11) years of age, using similar methods, providing the first opportunity to assess child–adult tracking in a LMIC. Ethical permission was obtained from the KEM Hospital Ethics Committee and informed consent was obtained from all the participants.

Weight was measured to the nearest 5 g, and height and waist circumference to the nearest 0.1 cm. Biceps, triceps, subscapular and suprailiac skinfolds were measured to the nearest 0.2 mm using calipers. Blood pressure was measured supine using a digital monitor; the average of two readings made 5 min apart was used for analysis. Fasting venous blood was drawn for plasma lipid, glucose, insulin and leptin measurements. An oral glucose tolerance test was performed giving 1.75 g/kg (8 years) and 75 g (21 years) anhydrous glucose in water, followed by a 120-minute blood sample (WHO protocol). Plasma glucose, cholesterol, HDL-cholesterol, and triglyceride concentrations were measured using standard enzymatic methods. Plasma insulin and leptin were measured using an immunoenzymometric assay and RIA respectively at 8 years, and a Delfia technique and ELISA at 21 years. Insulin resistance (HOMA-IR) was calculated at both ages using the online Oxford model (http://www.dtu.ox.ac.uk). Overweight was defined as BMI ≥ 25 kg/m^2^ and < 30 kg/m^2^, obesity as BMI ≥ 30 kg/m (WHO criteria) and central obesity as waist circumference ≥ 90 cm in men and ≥ 80 cm in women (IDF criteria). Hypertension was defined as systolic blood pressure ≥ 130 mm Hg or diastolic blood pressure ≥ 85 mm Hg (IDF criteria). Impaired fasting glucose (IFG) was defined as fasting glucose ≥ 100 mg/dl and < 126 mg/dl, impaired glucose tolerance (IGT) as 120-minute glucose ≥ 140 mg/dl and < 200 mg/dl, and diabetes mellitus (DM) as fasting glucose ≥ 126 mg/dl or 120-minute plasma glucose ≥ 200 mg/d (ADA criteria). Hypercholesterolaemia was defined as total cholesterol ≥ 200 mg/dl (NCEP criteria), hypertriglyceridaemia as triglycerides ≥ 150 mg/dl, and low HDL-cholesterol as HDL-cholesterol < 40 mg/dl for men and < 50 mg/dl for women (IDF criteria).

*Statistical methods*: We assessed tracking between the two ages using Pearson correlation coefficients. We categorized each risk factor by quartiles and calculated the relative risk of persisting in the highest or lowest categories between 8 and 21 years. We assessed the agreement between category status at the two ages using Cohen's kappa statistic. We calculated sensitivity, specificity, and positive and negative predictive values for being in the highest risk category at 8 years and disease outcomes at 21 years (overweight/obesity, central obesity, hypertension, dyslipidaemia, hyperglycemia). Statistical analyses were performed using SPSS 16.0.

Of 477 children studied at 8 years, 357 (75%) participated at 21 years (191 males). Non-participants had higher 8-year BMI (p = 0.05) and larger 8-year subscapular (p = 0.03), triceps (p = 0.02), and biceps (p = 0.05) skinfolds but similar values for all other measurements compared to participants. Of the participants, 18.5% were overweight, 2.5% were obese, 4.8% were hypertensive, 5.6% had hypercholesterolaemia, 7.6% had hypertriglyceridaemia, and 69.3% had low HDL-cholesterol at 21 years. Three participants were known to have diabetes; a further 18.5% were found to be hyperglycaemic (11.2% IFG, 5.9% IGT and 1.4% DM).

All the 8-year measurements were significantly positively correlated with their corresponding 21-year measurements, except 120-minute glucose concentration (Table 1); findings were similar in both sexes. Correlation coefficients for the age- and sex-adjusted values ranged from r = 0.53 (total cholesterol and BMI) to r = 0.14 (fasting insulin and HOMA-IR) (p < 0.05 for all). They were also similar using BMI-adjusted variables.

The risk of an individual being in the highest-risk category at 21 years was increased if they were in the same category at 8 years; these associations were strongest for cholesterol and BMI and weakest for fasting insulin, glucose and systolic blood pressure (Table 1). There was a similar tendency to persist in the lowest-risk category between 8 and 21 years. The strength of tracking showed similar results; the closest agreement between categories at 8 and 21 years was observed for cholesterol (Cohen's kappa = 0.36), while the weakest was observed for 120-minute glucose (Cohen's kappa = 0.06).

Positive predictive values of being in the high-risk group at 8 years for developing disease outcomes ranged from 73% (low HDL-cholesterol) to 8% (hypertension) (Table 2). Negative predictive values ranged from 98% (hypercholesterolaemia) to 32% (low HDL-cholesterol). Sensitivity values were less than 50% except for hypercholesterolaemia (75%). Specificity values were 70–80%.

These are the first data on child–adult tracking of a range of CVD risk factors from a LMIC. We found significant tracking for all risk factors, similar in both sexes. Measures of adiposity (BMI, skinfolds, waist circumference) and total cholesterol showed strong tracking. Intermediate tracking effects were seen for blood pressure, triglycerides, HDL-cholesterol, fasting glucose and insulin resistance while tracking was weakest for 120-minute glucose concentrations.

Our findings are consistent with studies from high-income countries [4–7]. Tracking may occur due to persistence of environmental factors through childhood and into adulthood. A meta-analysis showed that dietary behavior and physical activity tracks from childhood into adulthood [9]. As both are modifiable factors, it suggests intervening earlier in the lifecourse may be beneficial. Tracking may also be due to genetic factors. Another possible reason could be ‘programming’ of risk factors during early life, and may suggest stability of epigenetic changes established earlier. Our previous finding linking birthweight to insulin resistance at four years [10] supports this possibility adding weight to interventions before and during pregnancy to improve fetal growth.

Despite significant correlations of child and adult risk factors, predictive values do not support their use as screening tools. This suggests that measures to reduce risk factors in children should be public health interventions, such as encouraging regular physical activity and healthier diets, rather than individually targeted interventions.

## Figures and Tables

**Table 1:** Tracking of CVD parameters from childhood to young adulthood.

	8 to 21 years (n = 354)					
Age and sex specific variables	r (p-value)	% in highest fourth of the distribution at 8 who remained there at 21 y	Relative risk (95%CI)	% in lowest fourth of the distribution at 8 who remained there at 21 y	Relative risk (95% CI)	Cohen's kappa (p-value)
BMI	0.53 (< 0.001)	50.6	2.87 (2.05, 4.01)	47.7	2.86 (2.02, 4.05)	0.33 (< 0.001)
Height	0.44 (< 0.001)	43.4	2.43 (1.70, 3.46)	44.9	2.36 (1.68, 3.31)	0.23 (< 0.001)
Waist circumference	0.44 (< 0.001)	48.3	2.67 (1.91, 3.74)	40.9	2.12 (1.49, 3.02)	0.28 (< 0.001)
Sum of skinfolds	0.44 (< 0.001)	49.4	2.80 (2.00, 3.93)	47.7	2.73 (1.94, 3.85)	0.31 (< 0.001)
Systolic BP	0.21 (< 0.001)	31.3	1.37 (0.93, 2.02)	30.6	1.37 (0.93, 2.03)	0.07 (0.03)
Diastolic BP	0.28 (< 0.001)	35.3	1.64 (1.13, 2.38)	25.3	1.00 (0.66, 1.53)	0.14 (< 0.001)
Cholesterol	0.53 (< 0.001)	57.5	4.19 (2.94,5.97)	49.4	2.88 (2.05, 4.06)	0.36 (< 0.001)
Triglycerides	0.33 (< 0.001)	37.4	1.79 (1.25, 2.56)	40.2	2.07 (1.45, 2.96)	0.22 (< 0.001)
HDL-cholesterol	0.26 (< 0.001)	36.0	1.64 (1.14,2.35)	38.9	1.94 (1.36, 2.77)	0.18 (< 0.001)
Fasting glucose	0.17 (0.006)	32.2	1.47 (1.00,2.15)	31.0	1.37 (0.93, 2.01)	0.07 (0.03)
120-minute glucose	0.07 (0.15)	27.4	1.20 (0.79, 1.82)	30.5	1.30 (0.87, 1.93)	0.06 (0.06)
Fasting insulin	0.14 (0.009)	28.4	1.18 (0.79, 1.77)	32.1	1.46 (1.00, 2.15)	0.08 (0.02)
HOMA-IR	0.14 (0.006)	35.6	1.65 (1.14, 2.38)	33.7	1.58 (1.08, 2.32)	0.11 (0.002)
Leptin	0.24 (< 0.001)	40.5	1.99 (1.37, 2.87)	39.0	1.86 (1.27, 2.69)	0.23 (< 0.001)

BMI: body mass index; BP: blood pressure; IR: insulin resistance.

**Table 2:** Positive and negative predictive values, sensitivity and specificity of childhood risk categories for disease outcomes at young adulthood.

Upper fourth risk category at 8 years	Disease outcome at 21 years	Positive predictive value	Negative predictive value	Sensitivity	Specificity
BMI	Overweight/Obesity	42%	85%	48%	82%
Waist circumference	Central obesity	43%	82%	44%	81%
Systolic BP	Hypertension	8%	96%	43%	76%
Diastolic BP	Hypertension	8%	96%	43%	76%
Cholesterol	Hypercholesterolaemia	17%	98%	75%	78%
Triglycerides	Hypertriglyceridaemia	14%	94%	50%	76%
HDL-cholesterol	Low HDL	73%	32%	26%	78%
Fasting glucose	Hyperglycaemia	26%	83%	33%	77%

BMI: body mass index; BP: blood pressure.
